# Comparative evaluation of osseointegration among different surface modification techniques in dental implants

**DOI:** 10.6026/973206300222213

**Published:** 2026-04-30

**Authors:** Shivani Saxena, Asad Musa Mujawar, Swapnaneel Pradhan, Mohsen Azimuddin Mohammed, Padmakanth Mannava, Laxman Roy Chittaluri, Rubeena Naaz, Md Kafeel Ahmed

**Affiliations:** 1 Department of Periodontology, Rajarajeswari Dental College and Hospital, Bangalore, Karnataka, India; 2 Department of Prosthodontics and Implantology, Private Practitioner, Mazaya Dental Center Muhayil, Saudi Arabia; 3 Department of Prosthodontics, Geetanjali Dental and Research Institute, Udaipur, Rajasthan, India; 4 Department of Prosthodontics, Bangalore, Karnataka, India; 5 Department of Periodontology, Geetanjali Dental and Research Institute, Udaipur, Rajasthan, India; 6 Department of Oral and Maxillofacial Surgery, Mamata Dental College and Hospital, Khammam, Telangana, India; 7 Private Practitioner, Dental Surgeon, SRK Dental Clinic, Hyderabad, Telangana, India; 8 Department of Periodontology and Implantology, MNR Dental College and Hospital, Sangareddy, Hyderabad, Telangana, India

**Keywords:** Osseointegration, dental implants, surface modification, sandblasted large-grit acid-etched (SLA), hydroxyapatite-coated (HA), implant stability quotient (ISQ)

## Abstract

Optimal osseointegration determines long-term dental implant success, yet the comparative effectiveness of surface modifications (SLA, TPS, HA, machined) remains insufficiently defined despite extensive research. This prospective study evaluated 120 implants across 80 patients randomized to sandblasted large-grit acid-etched (SLA), titanium plasma-sprayed (TPS), hydroxyapatite-coated (HA) or machined surfaces, measuring ISQ, marginal bone loss and BIC at baseline, 6, 12 and 24 weeks. SLA demonstrated superior ISQ (78.42±4.21) and BIC (72.18±6.34%) at 24 weeks versus machined surfaces (p<0.05), with HA performing closely second; TPS showed intermediate results. SLA surface modification yielded the highest osseointegration parameters, minimizing bone loss while maximizing stability across follow-up intervals. This helps to advance implantology by confirming SLA's clinical superiority over alternatives, guiding surface selection to optimize treatment predictability and longevity.

## Background:

The use of dental implants has transformed the rehabilitation of edentulous patients, offering functional and aesthetic solutions that closely resemble natural dentition [1]. The principle of so-called osseointegration, initially defined as direct structural and functional interlocking between ordered living bone and the surface of a load-bearing implant, has remained the basis of successful implant therapy [2]. Incredible breakthroughs in surface modification mark the modern state of implantology, enhancing the biocompatibility of bone-implant contact and promoting rapid bone-implant integration [3]. The surface properties of dental implants are crucial to the quality and speed of osseointegration. The cellular adhesion, proliferation and differentiation of osteogenic cells at the implant surface are affected by the surface topography, chemical composition, surface energy and wettability [4]. Awareness of these elements has spurred the development of numerous surface modification methods to optimize the biological environment for bone formation and mineralization [5]. The first-generation implant technology uses machined or turned implant surfaces with a relatively smooth topography and low bone-anchorage surface area. Although clinically successful, these surfaces exhibit slower osseointegration kinetics than roughened surfaces [6]. A further breakthrough came with the introduction of sandblasted, large-grit acid-etched surfaces, which combined macroscopic roughness from sandblasting with microscopic roughness from acid treatment to produce the best surface topography for osteoblast attachment [7]. Another way of surface modification is titanium plasma-sprayed surfaces, where titanium particles are shot onto the implant surface, forming a porous coating that increases mechanical interlocking with bone tissue around the implant [8]. The method can greatly increase surface area and provide a three-dimensional structure that supports bone growth. Nonetheless, there have been fears about the possibility of particle detachment and the peri-implant complications [9]. Attention has been paid to bioactive coating, especially hydroxyapatite, because of its osteoconductive and chemical similarity to the mineral component of natural bone [10]. These coatings enhance direct chemical bonding between the implant surface and the bone tissue, which may hasten the initial stages of healing. However, degradation and delamination over time are also active areas of research [11]. Nanotechnology may be used to modify the surface of implants and recent studies have shown that nanoscale topography can significantly alter cellular response and tissue reactions [12]. Studies have found that controlled nanotube structures on anodized surfaces enhance osteoblast differentiation and bone formation relative to conventional surfaces [13]. Although the field has been widely researched, there are still few comparative studies that assess various surface modification methods in controlled clinical settings. The diversity of the study designs, evaluation parameters and follow-ups makes comparison between various surface technologies difficult [14]. Moreover, the extrapolation of in vitro and animal research results to clinical results needs to be systematically evaluated in humans [15]. Surface modification in relation to long-term implant survival remains a subject of scientific controversy. Although higher rates of osseointegration could be used to enable early loading regimens and improve patient satisfaction, the effect of surface properties on marginal bone maintenance and peri-implant tissue health warrants an in-depth assessment [16]. Therefore, it is of interest to establish differences in the parameters of osseointegration, such as implant stability, marginal bone levels and bone-to-implant contact, among SLA, TPS, HA-coated and machined surface implants over a 24w observation period.

## Materials and Methods:

The study was a prospective comparative clinical trial conducted at the Department of Prosthodontics and Oral Implantology from January 2022 to December 2023.

## Sample size calculation:

The sample size was determined based on prior research reporting a mean difference in implant stability quotient between surface modification groups of 5 units with a standard deviation of 6 units. The lowest required sample size was determined to be 28 implants per group, based on an alpha level of 0.05 and a statistical power of 80. There were 30 implants in each group across the four studies, accounting for dropouts and complications. A total of 120 dental implants were assigned.

## Participant selection:

Eighty dental implant candidates who needed dental implant rehabilitation were enlisted in this study. The inclusion criteria included: Age 25 to 65 years, sufficient bone volume to place the implants without grafting, no systemic disease affecting bone metabolism, no smokers or former smokers who had stopped smoking at least 6 months beforehand and good oral hygiene with full-mouth plaque scores under 25. The exclusion criteria were as follows: Uncontrolled diabetes mellitus, immunocompromised condition and radiation therapy to the head and neck area, periodontitis, bruxism, pregnancy or lactation, chronic use of bisphosphonates or corticosteroids and poor adherence to the follow-up protocols. A randomized computer-generated assignment to the test participants involved a random distribution of four types of implant surface:

[1] Group A (n=30): Sandblasted, large grit, acid-etched (SLA) surface implants.

[2] Group B (n=30): Titanium plasma-sprayed (TPS) surface implants.

[3] Group C (n=30): Hydroxyapatite-coated (HA) surface implants.

[4] Group D (n=30): Machined (turned) surface implants.

To eliminate confounding variables, all implants were made of titanium alloy (Grade 4), cylindrical, root-form and of standard dimensions (diameter: 4.0 mm, length: 10.0 mm) from the same manufacturer.

## Surgical protocol:

The implant surgeon was experienced to ensure standardization across all operations. After the administration of local anesthesia (2 percent lidocaine with 1:100,000 epinephrine), a mid-crestal incision was made and full-thickness mucoperiosteal flaps were raised. The procedure was carried out sequentially, following the manufacturer's instructions and using a lot of saline irrigation to avoid burning. The crestal bone level was used to insert implants with an insertion torque of 30-45 Ncm. Stability of the primary resonance was checked using resonance frequency analysis immediately after insertion. The cases were related and primary closure was performed without tension using 4-0 resorbable sutures. Every patient received uniform post-operative education and antibiotic prophylaxis (amoxicillin 500 mg three times daily for 5 days).

## Parameters of outcome assessment:

## Implant stability quotient (ISQ): 

The Osstell ISQ device was used to measure resonance frequency at baseline (immediately after placement) and at 6, 12 and 24 weeks. Buccal and lingual measurements were taken and the mean values were computed to analyze them.

## Marginal Bone Loss (MBL):

Baseline, 12-week and 24-week standardized periapical radiographs were taken in paralleling mode with customized film holders. The calibration of the digital imaging software was performed at the margins of the bone at the implant-abutment junction, from the initial bone-to-implant contact, on the mesial and distal sides. Each implant was evaluated by calculating mean values on both surfaces.

## Bone-to-Implant contact (BIC):

In a subsample of participants (n=10 per group) who had to have implants removed due to prosthetic complications unrelated to osseointegration, histomorphometric analysis was performed on retrieved implant specimens. BIC percentage was determined by dividing the linear distance of the bone immediately in touch with the implant surface by the total length of the implant surface multiplied by 100.

## Statistical analysis:

SPSS version 26.0 (IBM Corporation, Armonk, NY) was used to analyze the data. The descriptive statistics were presented as the mean and standard deviation for continuous variables and frequencies (percentages) for categorical variables. The Shapiro-Wilk test was used to test the normality of the data distribution. The comparison of continuous variables among the four groups was performed using one-way analysis of variance (ANOVA) and the Tukey post-hoc test was used to compare two variables in case significant differences were noted. The effects of changes in ISQ and MBL over time were analyzed using repeated-measures ANOVA. The categorical variables were subject to a chi-square test. The level of statistical significance was p<0.05.

## Results:

A total of 120 implants were placed in 80 patients who completed the 24-week follow-up period. No implants were lost during the study period. The demographic and clinical characteristics of participants were comparable across all groups with no statistically significant differences (Table 1 - see PDF). ISQ values demonstrated progressive increase across all groups throughout the observation period, reflecting ongoing osseointegration (Table 2 - see PDF). At baseline, no significant differences were observed among groups. However, significant intergroup differences emerged at 6 weeks and persisted through the 24-week evaluation. Post-hoc analysis at 24 weeks revealed that Group A (SLA) demonstrated significantly higher ISQ values compared to Group D (Machined) (p<0.001) and Group B (TPS) (p=0.008). Group C (HA) showed significantly higher values than Group D (p<0.001). No significant difference was observed between Groups A and C (p=0.324). Marginal bone loss was minimal across all groups, with progressive increases observed over the study period (Table 3 - see PDF). At 24 weeks, Group D (Machined) exhibited the highest mean marginal bone loss (0.89 ± 0.28 mm), while Group A (SLA) demonstrated the lowest values (0.42 ± 0.18 mm). Histomorphometric analysis of retrieved implant specimens revealed significant differences in bone-to-implant contact percentages among groups. SLA surfaces demonstrated the highest BIC values (72.18 ± 6.34%), followed by HA-coated surfaces (69.45 ± 6.89%), TPS surfaces (64.23 ± 7.12%) and machined surfaces (56.78 ± 8.45%). All pairwise comparisons between Group A and Group D and between Group C and Group D, were statistically significant (p<0.001). All 120 implants achieved successful osseointegration with 100% survival rate at 24 weeks. Minor complications included transient swelling in 12 patients (15%) and mild discomfort in 8 patients (10%), both of which resolved spontaneously within 1 week. No significant differences in complication rates were observed among groups (p=0.678).

## Discussion:

The current research has provided detailed comparative information on the outcomes of different methods for achieving the best results in the periosteum of the surface during the performance of dental implants. The results indicate a clear superiority of SLA and hydroxyapatite- coated surfaces over machined surfaces across various evaluation parameters, with the best performance recorded by SLA surfaces during the 24-week observation period. The measured improvement in implant stability quotient values with SLA surfaces is consistent with prior research showing the positive impact of joint microscopic and macroscopic roughness on bone response [1]. Macroroughness generated through the sandblasting process improves mechanical interlocking and microroughness generated by the acid-etching process maximizes cellular attachment and signaling [2]. All these surface features stimulate enhanced osteoblast differentiation and bone matrix deposition. The gradual rise in ISQ values across all groups indicates a biological shift from primary mechanical stability to secondary biological stability during osseointegration [3]. Nevertheless, there was a marked difference in the rate and extent of stability gain between the surface modification methods. The stabilization and ISQ values of SLA surfaces were shown to be higher and to stabilize earlier, indicating increased bone remodeling activity at the implant interface [4]. The implants with hydroxyapatite coating were also shown to have good osseointegration parameters, indicating they were second only to the SLA surfaces in most outcome measures. Osteoconductive properties of hydroxyapatite make it easy to bond bone directly by chemically interacting between the coating and the bone tissue around it [5]. The percentage bone-to-implant contact of 69.45% for the HA-coated implants is within the range reported in clinical trials [6]. The titanium plasma-sprayed surfaces exhibited intermediate performance, with osseointegration parameters better than those of machined surfaces but worse than those of SLA and HA-coated surfaces. Although TPS surfaces can significantly increase the surface area and porosity of the bone to support growth, their topography can be relatively uniform, creating unfavorable conditions for osteogenic cell behavior compared to that offered by hierarchically organized surfaces [7]. Conventional first-generation implant technology, with machined surfaces, exhibited the lowest osseointegration parameters at all-time points evaluated. The surface topography is relatively smooth, which limits mechanical interlocking and biological contact with surrounding tissues [8]. However, the group's 100 percent survival rate supports the idea that machined-surface implants remain clinically viable; through their integration kinetics may be prolonged. When assessing the level of bone loss, there was a marked intergroup difference, with SLA surfaces showing the best bone preservation at 24 weeks. Surface modification and marginal bone stability are both well-studied and, in most cases, roughened surfaces have been associated with positive maintenance of crestal bone [9]. The better results with SLA surfaces could indicate better bone quality and maturation in the coronal implant area. The histomorphometric results provide first-hand evidence of differences in osseointegration formation across surface modification techniques. The bone-to-implant contact percentage of 72.18% achieved with SLA surfaces is higher than the previously reported thresholds for successful osseointegration and indicates strong biological integration [10]. These results are consistent with the resonance frequency analysis data and the clinical superiority of the SLA surface technology. A variety of mechanisms can be used to elaborate on the differences in the results obtained during the process of osseointegration. Surface roughness determines protein adsorption, which in turn affects cellular adhesion and differentiation [11]. Surface energy and wettability values also define the initial response of blood to the implant surface, affecting fibrin clotting and wound-healing dynamics [12]. Internal validity of the present study is reinforced by the standardization of the study design, including similarity in surgical procedures, implant sizes and evaluation parameters. Inter-operator variability was removed as a confounding factor because single-operator surgical procedures were used and selection bias was reduced by random allocation [13]. Modern trends in implant surface technology continue to explore new ways to enhance osseointegration. Some applications of nanotechnology, such as titanium oxide nanotube and nanostructured surfaces, have shown promising results in preclinical investigations [14]. Future research should consider whether these newly developed technologies yield better results than existing methods of surface modification. Our findings, as applicable to clinical practice, indicate that preferential use of SLA surface implants in standard implant therapy, especially in cases where rapid integration of these implants is required, such as immediate or early loading regimens [15]. Hydroxyapatite-coated implants are also options, particularly in scenarios where bone quality is compromised and increased osteoconduction could be beneficial. The short follow-up period is a limitation of the study, as it is impossible to determine the long-term stability of osseointegration and the results of the prostheses. Also, the histomorphometric analysis was performed only on a group of individuals and the results might not be fully representative of the entire population under study. The patients who have a history of systemic diseases and smoking are excluded, which restricts the generalizability to medically compromised populations [16]. Extended follow-up studies are recommended to assess the sustainability of the present research's results regarding the application of surface modification procedures and to compare differences in long-term survival and success rates across the application of these techniques [17]. Also, the study of molecular indicators of bone formation and resorption has the potential to yield mechanistic insights into the biological mechanisms underlying differential bone integration responses [18].

## Conclusion:

SLA (sandblasted large-grit acid-etched) implants achieved the highest osseointegration, with superior stability, minimal marginal bone loss and greatest bone-to-implant contact, followed by hydroxyapatite-coated surfaces. Titanium plasma-sprayed surfaces performed intermediately between SLA/HA and machined implants, all achieving clinical success but with differing quantitative osseointegration profiles. This shows SLA surfaces as the preferred clinical choice and suggests that enhanced osseointegration may permit accelerated loading and more predictable implant rehabilitation.
